# Topical DNA aptamer therapeutics for the skin

**DOI:** 10.1016/j.omtn.2024.102299

**Published:** 2024-08-23

**Authors:** Simon C.C. Shiu, Andrew B. Kinghorn, Julian A. Tanner

**Affiliations:** 1 School of Biomedical Sciences, Li Ka Shing Faculty of Medicine, The University of Hong Kong, Hong Kong, P.R. China; 2 Advanced Biomedical Instrumentation Centre, Hong Kong Science Park, Shatin, New Territories, Hong Kong, P.R. China; 3 Materials Innovation Institute for Life Sciences and Energy (MILES), HKU-SIRI, Shenzhen, P.R. China

## Main text

Irmgard Förster and Günter Mayer, together with their respective collaborative teams, report in this issue on the development of the first-ever DNA aptamer-containing topical cream.^1^ They successfully treated allergic contact dermatitis (ACD) in mice through DNA aptamer-mediated inhibition of the chemokine CCL22, which blocks the recruitment of T cells, thereby reducing inflammation associated with ACD. ACD is an inflammatory skin condition affecting 16.5% of the population,^2^ caused by an immune response to specific allergens such as metals, fragrances, and preservatives found in common items such as cosmetics, jewelry, or cleaning products. Most allergic reactions originate from chemokine-mediated cell migration that recruits immune cells as a result of inflammation.^3^ Inhibition of immune cell recruitment is a key therapeutic target. The manuscript shows identification and application of a DNA aptamer to inhibit CCL22 from signaling immune cells to block ACD inflammation in mice. Being the first example of a therapeutic DNA aptamer topical cream, this publication is a pivotal moment in aptamer-based therapeutics and, more specifically, for ACD treatment.

To achieve their goals, they first demonstrated the therapeutic opportunity to block ACD inflammation by using contact hypersensitivity, sensitization, and challenge and response assays with wild-type and CCL22 knockout mice. By assessing allergic response through mouse ear thicknesses, the CCL22-deficient mice showed a mild swelling response when compared with the wild type, indicating the important role of CCL22 as a therapeutic target for treatment of contact hypersensitivity.

The authors used systematic evolution of ligands by exponential enrichment to select a novel DNA aptamer, AJ102, against the CCL22 chemokine. Surface plasmon resonance, fluorescence-activated cell sorting, and a cell migration assay were used to characterize aptamers and truncated variants, resulting in the highly sensitive and specific AJ102.29. To increase the half-life and resistance to renal clearance, a 5′-polyethylene glycol tail and 3′-dT cap were conjugated to create AJ102.29m.

To demonstrate AJ102.29m therapeutic activity *in vivo*, intraperitoneal injection was used and showed significant reduction of ear swelling after 2,4-dinitrofluorobenzene challenge. For *ex vivo* topical application, AJ102.29m was formulated in an amphiphilic DAC cream, commonly used by dermatologists. Application of topical AJ102.29m resulted in effective reduction of contact hypersensitivity symptoms and penetration of the skin. Microscopy of sections of mouse ears treated with Atto647-labeled AJ102.29m in DAC cream revealed penetration of the aptamer into the epidermis and dermis. Importantly, the inhibitory effect through topical application was greater than that of systemic application. Topical application for ACD treatment would be valuable as a non-invasive method that can be applied specifically to the affected area and reduce unwanted adverse effects.

This work pioneered the clinical application of therapeutic aptamers with several breakthroughs. First, the increased stability of DNA aptamers (relative to RNA aptamers) enabled use of far lower concentrations of aptamer in the formulation,^4^ which lowers costs and likely reduces adverse effects. Second, formulation into a cream for topical application was found to be more effective than intraperitoneal injection, supporting observations that DNA aptamers are small enough to penetrate and reach therapeutic targets through application of topical creams. Third, antibody-mediated inhibition of both CCL17 and CCL22 did not result in amelioration of contact hypersensitivity.^5^^,^^6^ This significant success, where antibodies had faced challenges yet aptamers had been successful, could be due to the higher affinity of the inhibitory aptamers to their targets or, potentially, because aptamers have superior skin penetration properties compared to antibodies. The study was conducted in mice, and the team showed that the AJ102.29m aptamer does not bind to human CCL22, indicating that this particular aptamer likely cannot be used directly for human ACD. Nevertheless, this proof of concept for ACD treatment shows a clear pathway and pipeline for developing a human ACD therapeutic topical cream. Indeed, effective topical delivery of aptamers opens many dermatological therapeutic opportunities, including for skin cancers, acne, rosacea, and psoriasis, among others. It is particularly exciting to further consider this in terms of DNA nanostructure-mediated control of aptamer therapeutic action in the skin. This promising proof-of-principal study opens up various possibilities for DNA aptamer-based topical therapeutics.

## Declaration of interests

The authors declare no competing interests.
